# Histogram analysis of absolute cerebral blood volume map can distinguish glioblastoma from solitary brain metastasis

**DOI:** 10.1097/MD.0000000000017515

**Published:** 2019-10-18

**Authors:** Jianhua Qin, Ying Li, Donghai Liang, Yuanna Zhang, Weicheng Yao

**Affiliations:** a School of Medicine, Qingdao University, Qingdao; b Department of Radiology, Rizhao Central Hospital, Rizhao, P. R. China; c Department of Radiology; d Department of Neurosurgery, The Affiliated Hospital of Qingdao University, China.

**Keywords:** brain metastases, glioblastoma, histogram analysis, MRI, perfusion

## Abstract

Glioblastoma multiforme (GBM) is difficult to be separated from solitary brain metastasis (sBM) in clinical practice. This study aimed to distinguish two entities by the histogram analysis of absolute cerebral blood volume (CBV) map.

From March 2016 to June 2018, 24 patients with GBM and 18 patients with sBM were included in this retrospective study. The enhancing area was first segmented on the post-contrast T1WI, then the segmentation was copied to the absolute CBV map and histogram analysis was finally performed. Unpaired *t* test was used to select the features that could separate two entities and receiving operating curve was used to test the diagnostic performance. Finally, a machine learning method was used to test the diagnostic performance combing all the selected features.

Six of 19 features were feasible to distinguish GBM from sBM (all *P* < .001), among which energy had the highest diagnostic performance (area under curve, 0.84; accuracy, 88%), while a machine learning method could improve the diagnostic performance (area under curve, 0.94; accuracy, 95%).

Histogram analysis of the absolute CBV in the enhancing area could help us distinguish GBM from sBM, in addition, a machine learning method with combined features is preferable. It is quite helpful in the condition that the biological nature of peritumoral edema could not separate these two entities.

## Abstract 2

Supplemental Digital Content is available in the text

## 1 Introduction

Glioblastoma multiforme (GBM) and brain metastases (BMs) are the most common malignant tumors in brain.^[1,2]^ Generally, GBM is solitary and BM is multifoci. However, sometimes it is difficult to distinguish GBM from solitary BM (sBM) by conventional MR imaging where two entities show a similar radiological appearance.^[3,4]^ Besides, previous study confirmed that patients with extracranial tumors are more likely to suffer from GBM than patients without.^[5]^ As clinical management and prognosis are quite different between two entities, it is always preferable to separate GBM from sBM before resorting to a biopsy, especially when the lesions were located in the dangerous regions of brain.^[6]^

Traditional dynamic susceptibility contrast perfusion weighted imaging (DSC-PWI) has been confirmed as a useful tool in distinguishing GBM and sBM, where two entities have different pathological changes in the peritumoral edema.^[7]^ The infiltrative edema around GBM was found to have a higher relative cerebral blood volume (rCBV) than the vasogenic edema around sBM. However, not all the GBMs have significant infiltrative edema,^[3,8]^ therefore, rCBV was helpless under this condition. Evaluation on enhancing area is an alternative in addition to peritumoral edema. While previous studies using the parameter of rCBV showed that the rCBV value of enhancing tumor was not applicable in distinguishing GBM from sBM.^[3,7,9]^ However, rCBV was a semi-quantitative parameter that has a large subjective bias,^[10]^ besides, the assessment based on mean value of the enhancing parts may not be a powerful enough method. Both of drawbacks may result in the disappointing results.

Bookend DSC-PWI is a new quantitative MR perfusion technique that could avoid subjective bias in assessing the perfusion conditions of tumors as possible.^[10]^ This technique could generate an absolute cerebral blood volume (aCBV) map for quantitative analysis.^[11]^ Histogram analysis that with more parameters is a more powerful and detailed evaluating method than mean value in tumoral radiology.^[12]^ In this retrospective article, we aimed to explore whether histogram analysis of aCBV map on the enhancing tumors could separate GBM from sBM.

## 2 Materials and methods

### 2.1 Study population

We retrospectively collected 65 patients with GBM and 37 patients with sBM in Rizhao Central Hospital from March 2016 to June 2018. The study was approved by the institutional review board of Rizhao Central Hospital. Written informed consents were already obtained from all patients when they had the MRI examinations. All examinations were performed in compliance with the Declaration of Helsinki. Diagnosis was confirmed by the histopathologic results of the lesions. One pathologist (with over 15 years’ experience in neuro-oncology) from Rizhao Central Hospital was responsible for the histopathological results. We excluded 41 patients with GBM and 19 patients with sBM for the following reasons:

1. no Bookend DSC-PWI sequences (n = 55);
2. unqualified imaging quality (n = 5).

Finally, 24 patients with GBM and 18 patients with sBM were enrolled in this study. The clinical characteristics of both groups are summarized in Table 1.

**Table 1:**
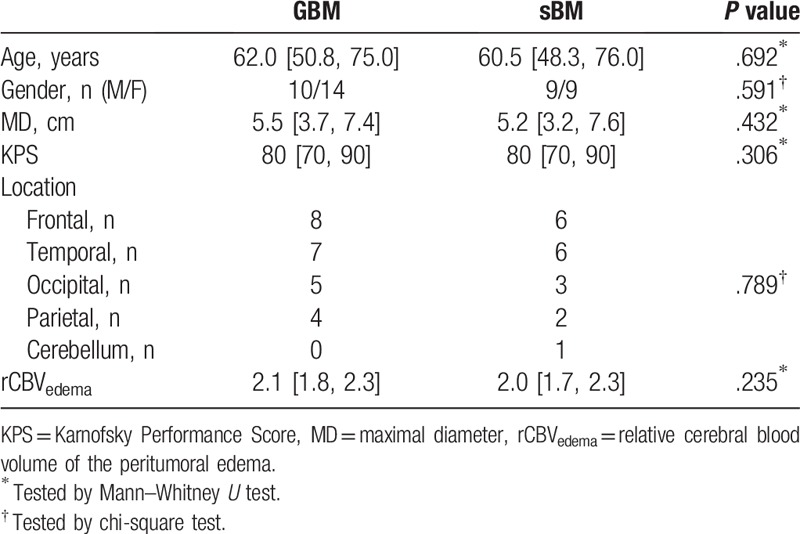
Clinical characteristics of the study population.

### 2.2 MRI protocol

All patients underwent MR scans on a 3T scanner (MAGNETOM Skyra, Siemens, Erlangen, Germany) using a 20-channel head-neck coil. The MR examinations of all included patients consisted conventional protocol for brain examination (T2WI, pre-contrast T1WI, and T2-FLAIR) and a prototype quantitative DSC-PWI sequence called ScalePWI. Then a post-contrast T1WI with same slice numbers and thickness to the ScalePWI was performed. The imaging parameters of post-contrast T1WI were as follows: repetition time/echo time 250 ms/2.5 ms, inversion time 900 ms, field of view (FOV) 220 × 220 mm, slice thickness 5 mm, and flip angle 70°. The imaging protocol was the same for all patients.

### 2.3 ScalePWI

A prototype quantitative DSC-PWI sequence named ScalePWI, which was provided by the Siemens Healthineers, was used in this study. The imaging parameters of ScalePWI were as follows: repetition time/echo time 1600 ms/30 ms, bandwidth 1748 Hz/pixel, 21 axial slices, FOV 220 × 220 mm, voxel size 1.8 × 1.8 × 4 mm^3^, slice thickness 5 mm, and flip angle 90°. For each slice, 50 measurements were acquired for each DSC-PWI analysis. After 46 s of injector delay, 0.2 mmol/kg bodyweight of contrast agent (Gd-DTPA, Magnevist; Schering, Berlin, Germany), followed by a 20 mL saline flush, was administered. An injection velocity of 4.5 mL/s was used in this study. Quantification of cerebral blood volume (CBV) is based on the Bookend technique,^[11]^ where the value of aCBV is dependent on the change of white matter before and after the injection of contrast agent:
 
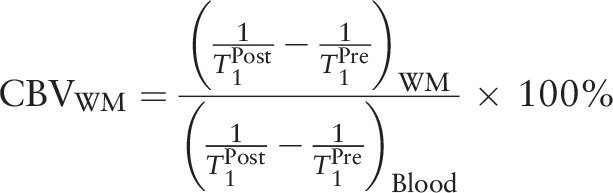


quantification of CBV_WM_:
 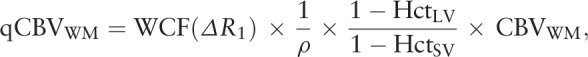


where 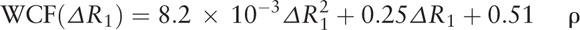, *Hct*_*LV*_, *Hct*_*sv*_, ΔR_1_ were all constant values. aCBV of each voxel was calculated by the following functions:
 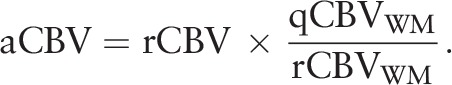


Unit of aCBV is mL/1000 g.

### 2.4 Segmentation for the enhancing area

The post-contrast T1WI images were conducted into a commercial-free software called *3D Slicer* (version 4.10, https://www.slicer.org/). Then the process of segmentation was performed by two neuroradiologists (with an experience of 10 years and 15 years separately) using the module of *Segment Editor*, where only the enhancing area of tumors were covered. The representative slice of each tumor for segmentation was selected in the principle of that with the largest tumoral diameter. Then the segmentations were saved and copied to the aCBV maps. Because both imaging sequences had the identical imaging protocol, the segmentations could be registered to the aCBV maps correctly. Both of neuroradiologists were blind to their segmentation results.

### 2.5 Histogram analysis

Both the aCBV maps and segmentation files were transferred to the module of *Radiomics*. Extraction Customization was selected as manual customization. Only the first order (corresponding to the histogram analysis) was selected in the feature classes. The first order included 19 features. The values of each feature from two neuroradiologists were averaged to represent features of each tumor. The analytic results of all tumors were collected in a new table. The detailed mathematical descriptions of first order were provided in Supplementary files (see Supplemental Methods, Supplemental Digital Content 1, which lists the detailed descriptions of First-order).

### 2.6 Feature selection of histogram analysis

An unpaired *t* test or Mann–Whitney *U* test was performed to select the features that had significant difference between two groups. Data of the selected features were first used to compute the diagnostic performance of each selected feature, then all the data was assigned to the training data to compute the diagnostic performance of combined features.

### 2.7 Diagnostic performance of combined features

**Figure 1:**
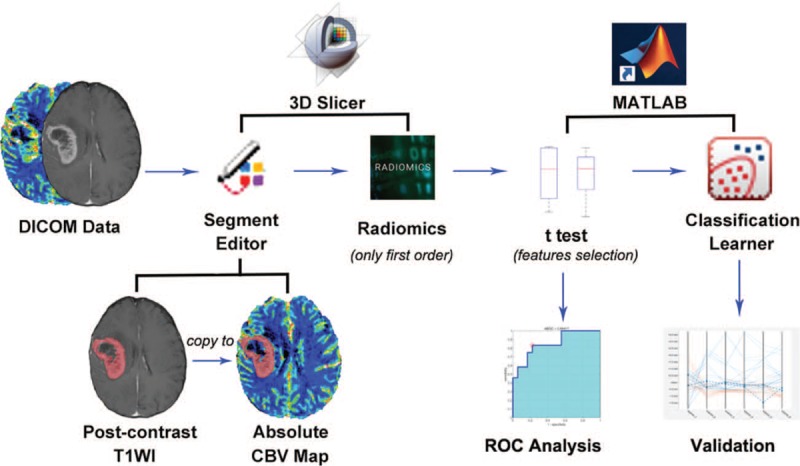
Illustration of the workflow in this study.

These steps were all performed in the commercially available software MATLAB (version 2018a, Mathwork, Inc, Natick, MA). Each feature of the training data was first normalized using the *mapminmax* function. Then the normalized training data was conducted into the toolbox of *Classification Learner*. Five kinds of training models were used to train the data one by one, including decision trees, linear discriminant, logistic regression, linear support vector machines (SVM), nearest neighbor classifiers (KNNs). Validation was performed by cross-validation with 5 folds, which could protect against overfitting by partitioning the data set into folds and estimating accuracy on each fold.^[13]^ Training model with the highest accuracy would be chosen to perform again using principal component analysis (PCA). The illustration of the workflow in this study is shown in Figure 1.

### 2.8 Statistical analysis

The Kolmogorov–Smirnov test was used to check the conditions of normal distribution of data. Mean ± standard deviation was used to describe the normally distributed variables and descriptive statistics (median and other measures) were used to describe the non-normally distributed variables. Differences in former variables were tested by unpaired *t* test and that in latter variables by Mann–Whitney *U* test. Differences in count variables were tested using chi-square test. Receiver operating curve (ROC) was performed to validate the diagnostic performance of features. Above steps were performed in MATLAB (version 2018a, Mathwork, Inc, Natick, MA). A *P* value < .05 was considered as significantly different.

## 3 Results

### 3.1 Study population

The information of study population is summarized in Table 1. No characteristics were found to be significantly different between two groups. The relative CBV of edematous area was also not significantly different (*P* = .235).

### 3.2 Histogram analysis and feature selection

**Figure 2:**
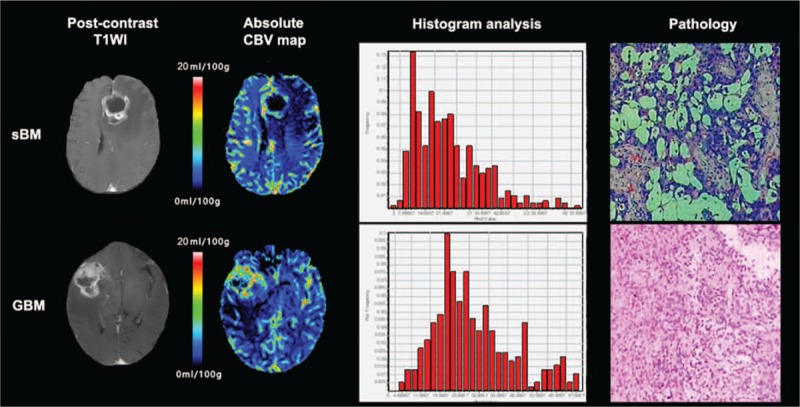
Examples of sBM and GBM and their results of histogram analysis. First row: a 76-year-old female patient was admitted to our hospital with a chief complaint of sensory and motor disturbance, post-contrast T1WI showed an enhancing tumor in the left frontal lobe, histogram analysis of absolute CBV in the enhancing area showed a left distribution, pathological result was sBM; second row: a 67-year-old male patient was admitted to our hospital with a chief compliant of motor disturbance and alalia, post-contrast T1WI showed an enhancing tumor in the right temporal lobe, histogram analysis of absolute CBV in the enhancing area showed a relatively normal distribution, pathological result was GBM. GBM = glioblastoma multiforme, sBM = solitary brain metastasis.

The results of histogram analysis are summarized in Table 2. Among the 19 features, 6 features were found to be significantly different between two groups. These features were skewness, median, energy, total energy, root mean squared, 10th percentile. The examples of sBM and GBM with histogram analysis are shown in Figure 2. The detailed information of patients’ clinical characteristics and results of histogram analysis are provided in Supplementary files (see Table S1, Supplemental Digital Content 2, which illustrates the histogram analysis results of patients with GBM; and see Table S2, Supplemental Digital Content 3, which illustrates the histogram analysis results of patients with sBM).

**Table 2:**
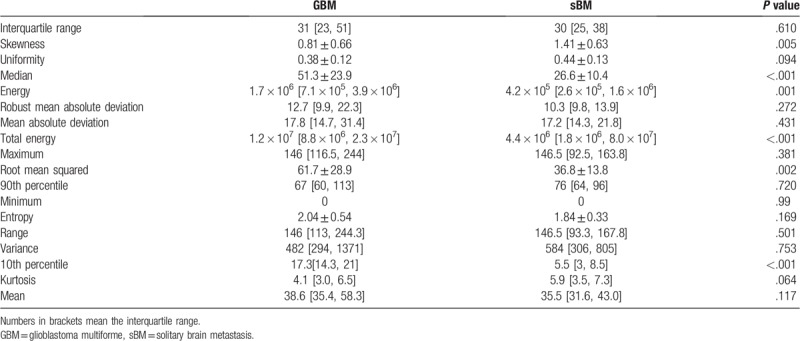
Results of histogram analysis in two groups.

### 3.3 Diagnostic performance of each selected feature

The diagnostic performances of each selected feature were collected in Table 3. Our results showed that the energy had the highest diagnostic performance, but the 10th percentile was similar to energy. Skewness had the worst diagnostic performance among the six features. The images of ROC were summarized in the Supplementary files (see Figure S1, Supplemental Digital Content 4, which demonstrates the ROC analysis results of each selected feature).

**Table 3:**
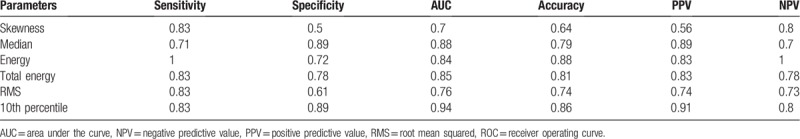
ROC curve analysis of each selected feature.

### 3.4 Training model selection and performance

**Figure 3:**
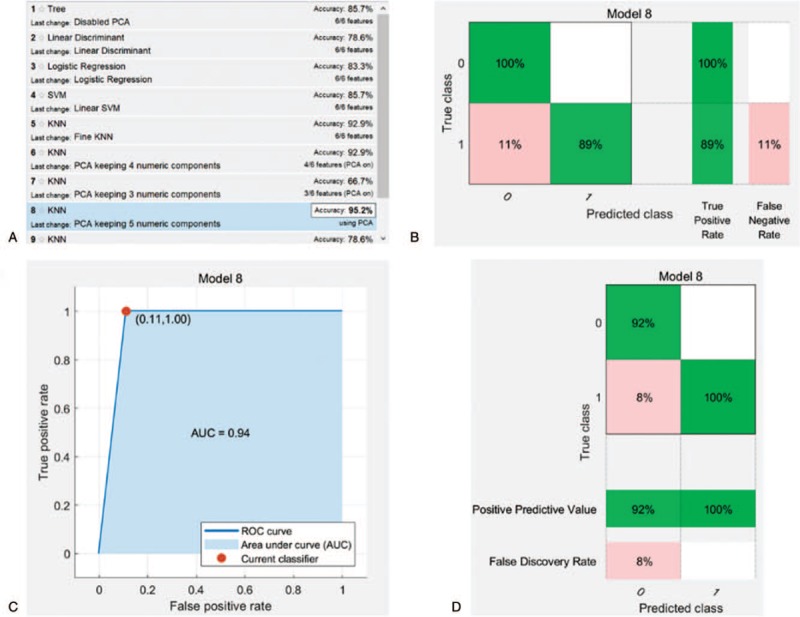
Results of diagnostic performance by machine learning method. (A) Results of different training models, KNN with PCA keeping 5 numeric components had the highest diagnostic accuracy; (B) true positive rate in predicting two entities; (C) ROC curve of the diagnostic performance of the best training model; (D) positive predictive value in predicting two entities.

Among the five training models, KNN got a highest accuracy of 92.9%. Accuracy of decision trees, linear discriminant, logistic regression and SVM were 85.7%, 78.6%, 83.3%, and 85.7%, separately. Adding PCA to KNN model improved the accuracy to 95.2%. Model type was as follows: Preset, fine KNN; Number of neighbors, 1; Distance weight, equal; Distance metric, Euclidean; Standardize data, true. There are 5 components kept in the PCA, in which explained variance per component (in order) were 99.1%, 0.9%, 0%, 0%, and 0%, separately. The area under curve was 0.94. The diagnostic performance is shown in Figure 3.

## 4 Discussion

Differential diagnosis between GBM and sBM has been a challenge in clinical practice.^[6]^ Although studies on the biological nature of peritumoral edema had improved the diagnostic efficiency,^[7,9]^ it is important to excavate more information from the enhancing tumors, because much infiltrative edemas around GBM was not specific enough compared with the vasogenic edema.

Our results showed that the mean value of aCBV of the enhancing tumor area failed to distinguish the two entities. Because previous studies had already confirmed the discouraging results of rCBV,^[7,9]^ our results further confirmed that mean values of both qualitative and quantitative CBV of the enhancing area would result in a discouraging result. However, the median value and 10th percentile of aCBV were feasible to distinguish the two entities, this result implies that the enhancing area of tumors is heterogenous that the extreme value prevents a reliable evaluation of the physiological conditions, but the histogram cutoff analysis not. As the median value and 10th percentile of aCBV in GBM is larger than that in sBM, this means the sBM has a relatively lower perfusion distribution in comparison with GBM. In clinical practice, the large sBM, which is hardly separated from GBM, usually comes from the lung cancer.^[14,15]^ The metastases from lung cancer were considered as low perfusion tumor until now.^[16]^ This may explain why the median value and 10th percentile of aCBV could distinguish the two entities.

Energy, total energy and root mean squared are three features that reflect the magnitude of the voxel in images. As these features of aCBV in GBM are larger than that in sBM, these results also imply that the GBM has a relatively higher perfusion than sBM. The reasons have been listed above.

Skewness measures the asymmetry of the distribution of values about the mean value. Skewness can be negative, positive, zero, or undefined according to the conditions of distribution. Skewness is also a feature that reflects the heterogeneity, this result implies that the sBM is more heterogeneous than GBM in the distribution of blood perfusion. The pathophysiological basis of this difference in blood perfusion may be that sBM has a much more different expression level of vascular epidermal growth factor in tumor area,^[17]^ in comparison with GBM.

Our results showed that the selected features had a good diagnostic performance, among which the energy and 10th percentile had the highest diagnostic performance. These results confirmed the most important physiological difference in the enhancing area of GBM and sBM is that the GBM has a relatively higher microvascular density than sBM.^[18]^ Despite the good performance of single feature, however, the combined features set could greatly improve the diagnostic performance (95.2% vs 88%), suggesting the training model of machine learning is a powerful tool in the differential diagnosis between GBM and sBM. For the further investigation about GBM and sBM, the machine learning method is suggested here.

Although features extraction in radiomics could provide hundreds of features, while we only selected the first order features. We made this decision for the following reasons:

1. the most important reason is that the features in first order are easy to interpret with the physiological process in tumors,^[19]^ other features including gray level co-occurrence matrix (GLCM) and gray level run length matrix (GLRLM) are difficult in interpretation;
2. the absolute CBV map has a much lower spatial resolution than the anatomical images (post-contrast T1WI, etc), this may result in an unsatisfied analytic result when using features influenced by voxel size.

Some limitations should be addressed here. First, this study only included a small sample size, that's because the incidence of large sBM was not very common in clinical practice, while in order to ensure the feasibility of validation process, a cross-validation of 5 folds was used in this study; secondly, whether the diagnostic performance of histogram analysis of absolute CBV map would improve when adding the radiomic features of anatomic images remains uncertain, we will perform this study and report the results in the future.

In conclusion, histogram analysis of absolute CBV in the enhancing area could help us distinguish GBM from sBM, among which six features were feasible in this differential diagnosis, besides, a machine learning method could improve the diagnostic performance. It is quite helpful in the condition that the biological nature of peritumoral edema could not separate these two entities.

## Author contributions

**Conceptualization:** Jianhua Qin, Weicheng Yao.

**Data curation:** Ying Li, Yuanna Zhang.

**Formal analysis:** Yuanna Zhang.

**Investigation:** Ying Li.

**Methodology:** Donghai Liang, Weicheng Yao.

**Software:** Jianhua Qin.

**Supervision:** Yuanna Zhang.

**Validation:** Donghai Liang.

**Visualization:** Yuanna Zhang.

**Writing – original draft:** Jianhua Qin.

**Writing – review & editing:** Jianhua Qin, Weicheng Yao.

## Supplementary Material

Supplemental Digital Content
