# Supplementary material for: Histogram analysis of absolute cerebral blood volume map can distinguish glioblastoma from solitary brain metastasis
Source: Medicine (Baltimore). 2019 Oct 18;98(42):e17515. doi: 10.1097/MD.0000000000017515 (PMC6824738; doi:10.1097/MD.0000000000017515)

**Supplemental Methods.** **Detailed descriptions of First-order**

First-order statistics describe the distribution of voxel intensities within the image region defined by the mask through commonly used and basic metrics.

Let:

- **X** be a set of *Np* voxels included in the ROI
- **P**(*i*) be the first order histogram with *Ng* discrete intensity levels, where *Ng* is the number of non-zero bins, equally spaced from 0 with a width defined in the bin Width parameter.
- *p*(*i*) be the normalized first order histogram and equal to **P**(*i*)/*Np*.

(**1) Energy**


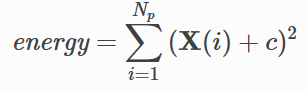


Here, *c* is optional value, defined by voxel Array Shift, which shifts the intensities to prevent negative values in **X**. This ensures that voxels with the lowest gray values contribute the least to Energy, instead of voxels with gray level intensity closest to 0.

Energy is a measure of the magnitude of voxel values in an image. A larger value implies a greater sum of the squares of these values.

**(2) Total Energy**


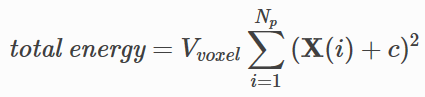


Here, c is optional value, defined by voxel Array Shift, which shifts the intensities to prevent negative values in X. This ensures that voxels with the lowest gray values contribute the least to Energy, instead of voxels with gray level intensity closest to 0.

Total Energy is the value of Energy feature scaled by the volume of the voxel in cubic mm.

This feature is volume-confounded, a larger value of cc increases the effect of volume-confounding.

**(3) Entropy**


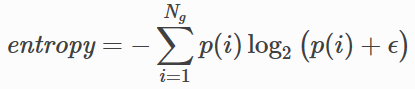


Here, ϵ is an arbitrarily small positive number (≈2.2×10^-16^).

Entropy specifies the uncertainty/randomness in the image values. It measures the average amount of information required to encode the image values.

**(4) Minimum**


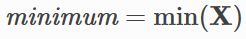


**(5) 10th percentile**

The 10th percentile of X

**(6) 90th percentile**

The 90th percentile of X

**(7) Maximum**


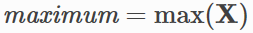


The maximum gray level intensity within the ROI.

**(8) Mean**


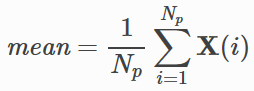


The average gray level intensity within the ROI.

**(9) Median**

The median gray level intensity within the ROI.

**(10) Interquartile Range**


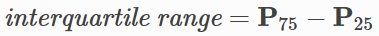


Here **P**_25_ and **P**_75_ are the 25^th^ and 75^th^ percentile of the image array, respectively.

**(11) Range**


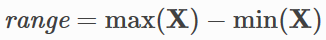


The range of gray values in the ROI.

**(12) Mean Absolute Deviation (MAD)**


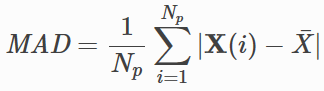


Mean Absolute Deviation is the mean distance of all intensity values from the Mean Value of the image array.

**(13) Robust Mean Absolute Deviation (rMAD)**


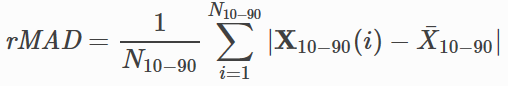


Robust Mean Absolute Deviation is the mean distance of all intensity values from the Mean Value calculated on the subset of image array with gray levels in between, or equal to the 10th and 90th percentile.

**(14) Root Mean Squared (RMS)**


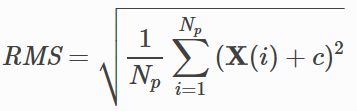


Here, c is optional value, defined by voxel Array Shift, which shifts the intensities to prevent negative values in X. This ensures that voxels with the lowest gray values contribute the least to RMS, instead of voxels with gray level intensity closest to 0.

RMS is the square-root of the mean of all the squared intensity values. It is another measure of the magnitude of the image values. This feature is volume-confounded, a larger value of c increases the effect of volume-confounding.

**(15) Standard Deviation**


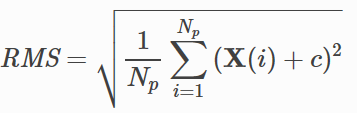


Standard Deviation measures the amount of variation or dispersion from the Mean Value. By definition,
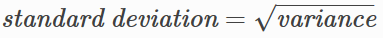


**(16) Skewness**


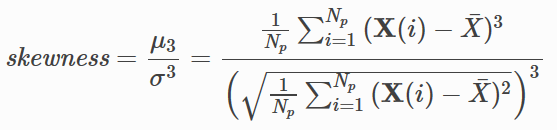


Where μ_3_ is the 3^rd^ central moment.

Skewness measures the asymmetry of the distribution of values about the Mean value. Depending on where the tail is elongated and the mass of the distribution is concentrated, this value can be positive or negative.

**(17) Kurtosis**


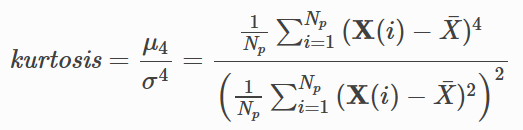


Where μ_4_ is the 4^th^ central moment.

Kurtosis is a measure of the ‘peakedness’ of the distribution of values in the image ROI. A higher kurtosis implies that the mass of the distribution is concentrated towards the tail(s) rather than towards the mean. A lower kurtosis implies the reverse: that the mass of the distribution is concentrated towards a spike near the Mean value.

**(18) Variance**


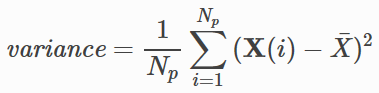


Variance is the mean of the squared distances of each intensity value from the Mean value. This is a measure of the spread of the distribution about the mean. By definition,
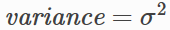


**(19) Uniformity**


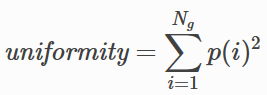


Uniformity is a measure of the sum of the squares of each intensity value. This is a measure of the homogeneity of the image array, where a greater uniformity implies a greater homogeneity or a smaller range of discrete intensity values.

**Table S1. Table of histogram analysis results of patients with GBM**

| **Age** | **Sex** | **InterquartileRange** | **Skewness** | **Uniformity** | **Median** | **Energy** | **RobustMeanAbsoluteDeviation** | **MeanAbsoluteDeviation** | **TotalEnergy** | **Maximum** | **RootMeanSquared** | **90^th^ Percentile** | **Minimum** | **Entropy** | **Range** | **Variance** | **10^th^ Percentile** | **Kurtosis** | **Mean** |
| --- | --- | --- | --- | --- | --- | --- | --- | --- | --- | --- | --- | --- | --- | --- | --- | --- | --- | --- | --- |
| 63 | F | 27.00 | 0.92 | 0.37 | 28 | 1765598 | 11.66 | 15.89 | 9181107 | 139 | 36.41 | 58 | 0.00 | 1.60 | 139 | 400 | 7.00 | 4.60 | 30.41 |
| 63 | M | 73.00 | 0.52 | 0.20 | 77 | 9616183 | 29.82 | 40.53 | 39059836 | 290 | 94.36 | 125 | 0.00 | 2.91 | 290 | 2460 | 16.90 | 3.04 | 42.27 |
| 60 | F | 81.00 | 0.75 | 0.24 | 85 | 4375624 | 34.23 | 46.63 | 71093207 | 267 | 111.97 | 137 | 0.00 | 2.78 | 267 | 3217 | 35.00 | 2.95 | 67.54 |
| 60 | F | 31.00 | 1.59 | 0.32 | 30 | 678104 | 12.54 | 19.23 | 11017529 | 147 | 44.27 | 67 | 0.00 | 1.87 | 147 | 653 | 10.00 | 6.66 | 36.14 |
| 50 | F | 49.75 | 1.12 | 0.26 | 67 | 1601830 | 21.78 | 32.06 | 26025801 | 280 | 82.74 | 95 | 1.00 | 2.67 | 279 | 1808 | 19.00 | 5.45 | 60.97 |
| 47 | M | 18.00 | 1.06 | 0.47 | 25 | 595910 | 7.50 | 10.36 | 3098731 | 101 | 29.09 | 42 | 0.00 | 1.20 | 101 | 184 | 9.00 | 6.40 | 25.72 |
| 75 | F | 47.00 | -0.30 | 0.21 | 92 | 4667363 | 19.19 | 26.86 | 24270300 | 169 | 94.92 | 108 | 2.00 | 2.46 | 167 | 1108 | 43.00 | 2.73 | 63.89 |
| 28 | M | 18.00 | 1.58 | 0.39 | 31 | 1544543 | 8.43 | 14.13 | 8031622 | 133 | 40.47 | 60 | 0.00 | 1.65 | 133 | 383 | 17.00 | 6.56 | 35.41 |
| 66 | F | 21.00 | 1.28 | 0.37 | 31 | 708875 | 9.53 | 14.63 | 11517492 | 145 | 41.08 | 60 | 0.00 | 1.67 | 145 | 376 | 17.00 | 6.20 | 36.21 |
| 66 | M | 31.00 | 0.12 | 0.30 | 49 | 2302040 | 12.92 | 18.11 | 11970605 | 112 | 54.33 | 79 | 0.00 | 1.93 | 112 | 513 | 21.00 | 2.79 | 49.38 |
| 26 | F | 24.00 | -0.28 | 0.38 | 40 | 602800 | 9.91 | 13.03 | 3134560 | 78 | 41.68 | 58 | 4.00 | 1.53 | 74 | 249 | 15.00 | 2.40 | 38.57 |
| 62 | F | 20.00 | 0.52 | 0.41 | 34 | 974109 | 8.63 | 12.40 | 15826895 | 100 | 38.02 | 56 | 0.00 | 1.48 | 100 | 254 | 15.00 | 3.56 | 34.51 |
| 63 | M | 31.00 | 1.05 | 0.43 | 32 | 1976483 | 13.42 | 17.47 | 9773504 | 143 | 42.72 | 62 | 0.00 | 1.74 | 144 | 450 | 9.00 | 4.72 | 30.41 |
| 62 | F | 78.00 | 0.64 | 0.27 | 81 | 9938366 | 32.43 | 43.62 | 41638175 | 298 | 102.54 | 124 | 0.00 | 2.86 | 298 | 1457 | 17.50 | 3.26 | 60.27 |
| 68 | F | 85.00 | 0.87 | 0.19 | 88 | 4663866 | 36.71 | 49.31 | 73175785 | 288 | 123.53 | 155 | 0.00 | 2.91 | 275 | 2456 | 38.00 | 3.11 | 56.54 |
| 53 | M | 36.00 | 1.73 | 0.44 | 34 | 727549 | 14.62 | 22.22 | 12374582 | 164 | 48.84 | 72 | 0.00 | 2.18 | 162 | 713 | 14.00 | 6.94 | 36.14 |
| 62 | M | 52.53 | 1.33 | 0.35 | 72 | 1735648 | 24.85 | 35.93 | 15734986 | 293 | 88.19 | 114 | 1.00 | 2.58 | 284 | 1540 | 21.00 | 5.77 | 70.97 |
| 32 | M | 22.00 | 1.26 | 0.61 | 29 | 624084 | 9.88 | 13.27 | 4057538 | 124 | 32.57 | 47 | 0.00 | 1.36 | 113 | 194 | 12.00 | 6.68 | 25.72 |
| 72 | F | 52.00 | -0.21 | 0.37 | 96 | 5247516 | 22.43 | 29.24 | 16585472 | 175 | 97.25 | 116 | 2.00 | 2.62 | 176 | 988 | 39.00 | 3.05 | 58.89 |
| 68 | M | 23.00 | 1.77 | 0.49 | 37 | 1738684 | 9.25 | 16.13 | 9154856 | 158 | 44.34 | 66 | 1.00 | 1.77 | 151 | 421 | 20.00 | 6.83 | 35.41 |
| 49 | F | 25.00 | 1.51 | 0.51 | 35 | 736583 | 9.89 | 17.55 | 8736982 | 161 | 47.55 | 64 | 0.00 | 1.84 | 163 | 395 | 21.00 | 6.53 | 36.21 |
| 73 | F | 34.00 | 0.29 | 0.42 | 53 | 2413860 | 14.27 | 21.32 | 9364713 | 135 | 58.98 | 84 | 0.00 | 2.06 | 128 | 546 | 23.00 | 3.04 | 49.38 |
| 58 | M | 36.00 | -0.24 | 0.53 | 47 | 654970 | 11.24 | 15.45 | 4259372 | 93 | 45.54 | 64 | 0.00 | 1.74 | 93 | 264 | 17.00 | 2.77 | 38.57 |
| 55 | F | 24.00 | 0.66 | 0.62 | 38 | 1037935 | 10.66 | 14.87 | 18754373 | 114 | 39.21 | 62 | 0.00 | 1.63 | 113 | 266 | 19.00 | 3.66 | 44.51 |

**Table S2. Table of** **histogram analysis results of patients with sBM**

| **Age** | **Sex** | **Skewness** | **Uniformity** | **Median** | **Energy** | **RobustMeanAbsoluteDeviation** | **MeanAbsoluteDeviation** | **TotalEnergy** | **Maximum** | **RootMeanSquared** | **90^th^ Percentile** | **Minimum** | **Entropy** | **Range** | **Variance** | **10^th^ Percentile** | **Kurtosis** | **Mean** |
| --- | --- | --- | --- | --- | --- | --- | --- | --- | --- | --- | --- | --- | --- | --- | --- | --- | --- | --- |
| 46 | F | 0.81 | 0.51 | 18 | 398888 | 9.83 | 13.23 | 2074219 | 76 | 26 | 63 | 0.00 | 2.19 | 76 | 259 | 3 | 2.99 | 40.71 |
| 64 | M | 2.05 | 0.49 | 14 | 458886 | 9.68 | 16.81 | 2386208 | 134 | 32 | 103 | 0.00 | 2.26 | 134 | 576 | 4 | 7.35 | 35.23 |
| 75 | M | 2.29 | 0.40 | 28 | 273482 | 9.97 | 15.34 | 4443413 | 152 | 38 | 122 | 0.00 | 1.54 | 152 | 490 | 8 | 10.13 | 30.81 |
| 41 | M | 0.87 | 0.30 | 29 | 1702384 | 12.64 | 19.29 | 8852391 | 147 | 49 | 102 | 0.00 | 1.94 | 147 | 649 | 12 | 4.50 | 52.02 |
| 49 | F | 1.32 | 0.32 | 33 | 2015065 | 12.99 | 19.66 | 10478346 | 192 | 45 | 78 | 0.00 | 1.88 | 192 | 691 | 6 | 6.46 | 46.44 |
| 76 | F | 0.88 | 0.46 | 21 | 266331 | 10.20 | 14.34 | 1081807 | 96 | 30 | 80 | 0.00 | 1.31 | 96 | 316 | 5 | 3.40 | 34.48 |
| 61 | F | 1.48 | 0.62 | 14 | 137343 | 4.73 | 7.11 | 557872 | 62 | 18 | 47 | 0.00 | 1.59 | 62 | 99 | 4 | 6.28 | 25.19 |
| 67 | M | 0.97 | 0.33 | 26 | 407280 | 21.98 | 27.72 | 6617308 | 155 | 46 | 75 | 0.00 | 1.92 | 155 | 1107 | 3 | 3.57 | 41.84 |
| 68 | M | 2.24 | 0.32 | 31 | 2379296 | 16.53 | 23.21 | 12372341 | 357 | 49 | 74 | 0.00 | 1.96 | 357 | 1088 | 2 | 10.10 | 36.07 |
| 59 | M | 0.64 | 0.20 | 37 | 346011 | 22.14 | 30.50 | 5621835 | 146 | 69 | 110 | 0.00 | 2.43 | 146 | 1333 | 17 | 2.42 | 68.37 |
| 45 | F | 0.95 | 0.62 | 20 | 476524 | 9.67 | 14.35 | 2255572 | 82 | 27 | 65 | 0.00 | 1.32 | 81 | 276 | 5 | 3.21 | 35.71 |
| 55 | F | 2.43 | 0.68 | 17 | 468954 | 9.81 | 17.51 | 2175126 | 145 | 35 | 86 | 0.00 | 1.46 | 145 | 593 | 3 | 7.14 | 33.23 |
| 71 | F | 2.57 | 0.52 | 31 | 254437 | 10.12 | 16.27 | 4354321 | 163 | 39 | 94 | 0.00 | 1.67 | 166 | 504 | 11 | 10.43 | 30.81 |
| 63 | M | 0.91 | 0.38 | 42 | 1504647 | 12.85 | 19.77 | 7743573 | 154 | 12 | 74 | 0.00 | 2.01 | 159 | 664 | 10 | 5.50 | 52.02 |
| 44 | F | 1.45 | 0.43 | 32 | 1814655 | 12.76 | 21.32 | 9969454 | 203 | 46 | 70 | 0.00 | 2.19 | 212 | 711 | 8 | 7.23 | 36.44 |
| 74 | M | 0.95 | 0.45 | 23 | 245353 | 10.32 | 15.83 | 1173629 | 105 | 33 | 53 | 0.00 | 1.55 | 108 | 332 | 7 | 4.26 | 34.48 |
| 66 | M | 1.56 | 0.57 | 15 | 157371 | 5.15 | 9.12 | 656955 | 77 | 20 | 59 | 0.00 | 1.77 | 85 | 115 | 6 | 6.86 | 28.19 |
| 54 | F | 1.05 | 0.45 | 23 | 428345 | 22.75 | 29.29 | 7238435 | 166 | 48 | 77 | 0.00 | 2.15 | 173 | 1232 | 3 | 4.12 | 31.84 |

**Figure S1. Figures of ROC analysis results.**


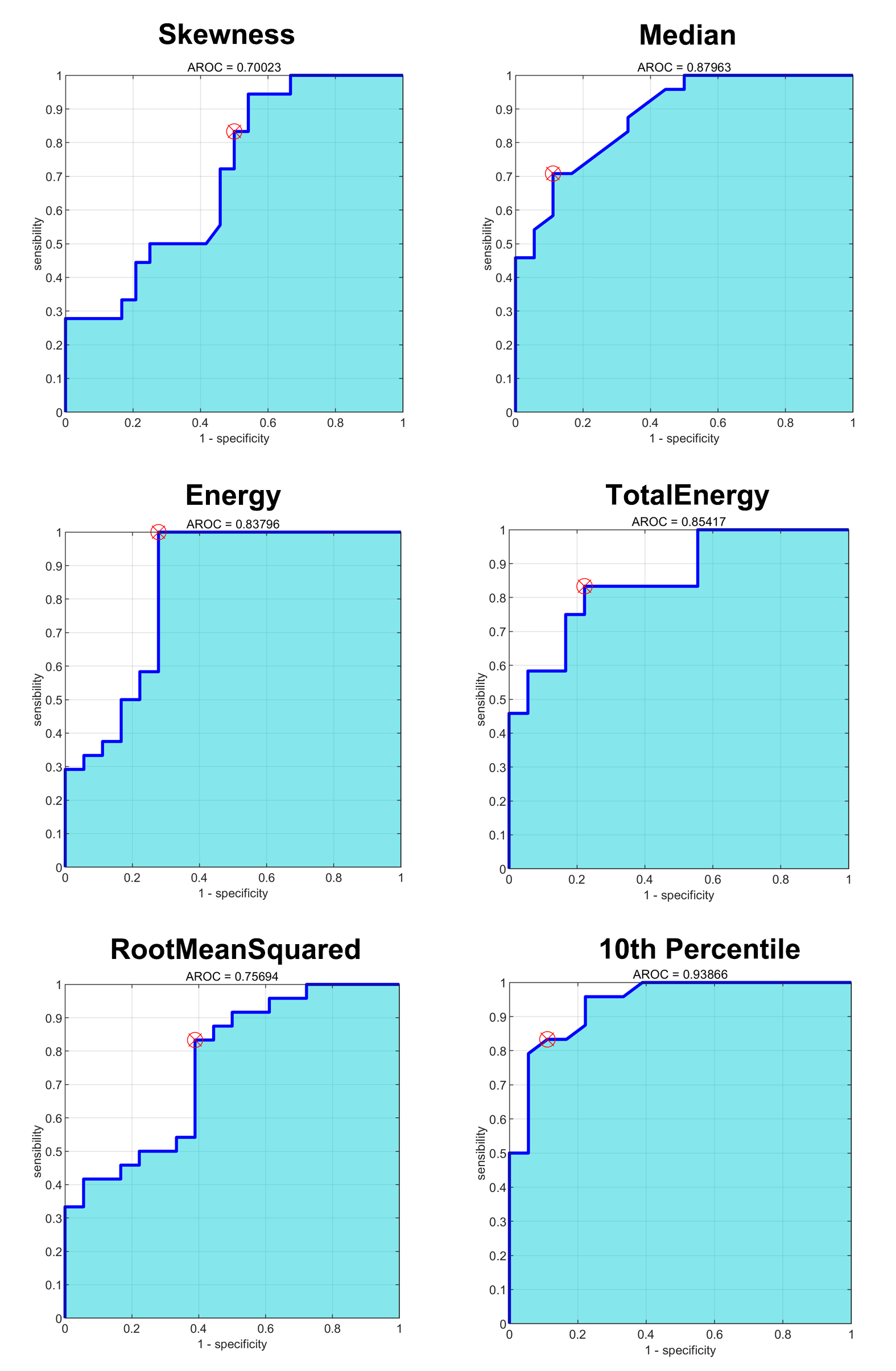

Supplement: Supplemental Digital Content [file medi-98-e17515-s001.docx]
